# Electron transfer and energy exchange between a covalent organic framework and CuFeS_2_ nanoparticles†

**DOI:** 10.1039/d4tc01989j

**Published:** 2024-06-14

**Authors:** Panagiota Bika, Vasileios K. Tzitzios, Elias Sakellis, Spyros Orfanoudakis, Nikos Boukos, Saeed M. Alhassan, Polychronis Tsipas, Vasileios Psycharis, Thomas Stergiopoulos, Panagiotis Dallas

**Affiliations:** a Institute of Nanoscience and Nanotechnology, NCSR Demokritos 15341 Athens Greece t.stergiopoulos@inn.demokritos.gr p.dallas@inn.demokritos.gr +302106503394 +302106503311; b School of Applied Mathematical and Physical Sciences, National Technical University Athens 15780 Zografou Athens Greece; c Department of Chemical Engineering, Khalifa University of Science and Technology P.O. Box 127788 Abu Dhabi United Arab Emirates; d National Institute of Materials Physics Atomistilor 405A Magurele Romania

## Abstract

CuFeS_2_ is a prominent chalcogenide that possesses similar optical properties and a significantly lower cost, compared to gold. Additionally, covalent organic frameworks are a class of materials at the forefront of current research, mainly used as photoactive components and porous absorbers. Hence, in this work, hydrophilic CuFeS_2_ particles are coupled with multi-functional covalent organic frameworks through ionic bonding to produce a hybrid material with unique and optimized properties. To render the CuFeS_2_ particles negatively charged and dispersible in water, we coated them with sodium dodecyl sulfonate, shifting the surface plasmon resonance of the nanoparticles from 472 to 492 nm. When they are electrostatically assembled with the positively charged COFs, an S-scheme is formed and the fluorescence of the hybrid materials is highly quenched, with the electron transfer happening from the networks to the nanoparticles and a simultaneous energy exchange which is dependent on the emission wavelength. Through detailed fluorescence spectroscopy, time-resolved measurements and Stern–Volmer analysis, we identified an efficient emission quenching that differs from the bulk to the exfoliated hybrid system, while detailed electron microscopy studies demonstrated the strong interaction between the two components. The quenching mechanisms and the on or off surface resonance dependent lifetime could be applied to photocatalytic and photovoltaic applications.

## Abstract 2

The graphic shows the structural representation of the electrostatic assembly of oppositely charged CuFeS_2_ nanoparticles with COF layers. Their addition in the COFs is enhancing the absorption capacity, while quenching the photoluminescence.

## Graphical abstract

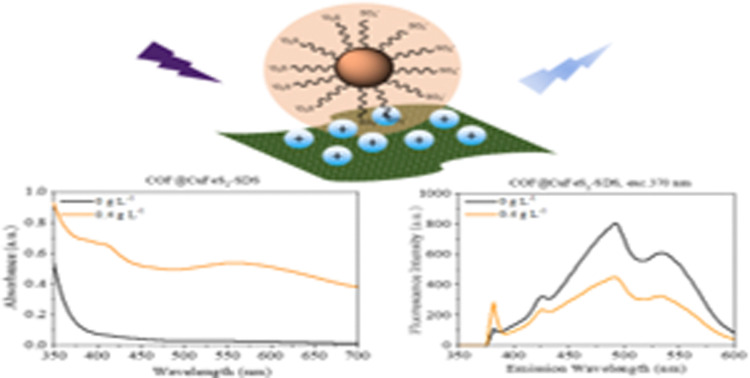

## Introduction

Nowadays, it is crucial to move towards earth-abundant, non-toxic, and eco-friendly materials that are also economically favourable. Sustainability targets set by the global community demand efficient recycling and utilization of all materials when possible. As an example, a huge excess of sulfur, the fifth most abundant element on Earth, is one of the main by-products of the petroleum industry. The majority of sulfur is used in making sulfuric acid, which is the largest produced chemical in the world. Aside from this application, sulfur is not used in other high-volume chemical industries. Consequently, sulfur valorisation, by converting it into other valuable products, is highly desirable. The conversion of elemental sulfur into novel nanomaterials is an alternative and prime solution. Among many materials with technological interest, the family of I–III–VI_2_ compounds with a ternary (ABX_2_) structure exhibit semiconducting properties suitable for optoelectronic and photocatalytic applications.^1,2^ CuFeS_2_, commonly known as the mineral chalcopyrite, has already attracted the interest of the research community.^3^ The bulk CuFeS_2_ mineral crystallizes at a tetragonal structure and has an optical indirect band gap of 0.5–0.6 eV, which can be modified by reducing its size to the nanometer range.^4^ It is a promising thermoelectric material with low resistivity and thermal conductivity and possesses an inter-band between conduction and valence band, predominately due to the vacant 3d orbitals of iron.^5^ CuFeS_2_ has intriguing optical, electrical, and magnetic properties and is an ideal alternative for non-noble metals plasmonic and especially for gold. It exhibits a prominent plasmonic band at 500 nm,^6^ attracting attention in the fields of photocatalysis,^1,7^ perovskite solar cells,^8^ sensors,^9^ thermoelectric applications,^10,11^ photothermal and photodynamic therapy.^5,12^

Furthermore, hybrid systems of 0D plasmonic nanomaterials with 3D and 2D semiconductors are creating several types of heterojunction systems^13^ and have new features since additional pathways across the interface become available for the generation of charge carriers and the subsequent energy conversion. Four types of transfer mechanisms from plasmonic nanoparticles to the semiconductor are determined: light scattering, light concentration, hot electron injection and plasmon-induced resonance transfer.^14^ Depending on the studied system, fluorescence quenching, enhancement or both are reported.^15,16^ In continuation, radiative or non-radiative pathways and static or dynamic quenching occur,^17^ impacting the lifetimes of the carriers.^16^ Exciton–plasmonic interactions are influenced by the direct contact or the distance between the two components of the hybrid materials,^18^ as well as whether there is resonant or non-resonant coupling.^19^ Consequently, there are several factors to consider when developing the different responses in hybrid materials.

Covalent organic frameworks (COFs) belong to a versatile class of materials that have found application in energy harvesting materials, heavy metal absorbers and photocatalysis, among others.^20,21^ They are great supports for anchoring nanoparticles, without post-treatment methods, concerning their already existing functional groups, heteroatoms, available lone electron pairs and π-conjugated system.^22,23^ For the *in situ* confinement of nanoparticles (NPs) to COFs, bottom-up and post-synthetic modification methods can be followed.^22,23^ Already, a lot of references have been reported for conventional plasmonic nanoparticles such as CdS, Ag, and Au with COFs for photocatalytic^24,25^ and sensing applications.^26,27^

In this work, a new method is reported for the synthesis of spherical CuFeS_2_ plasmonic nanoparticles, using elemental sulfur as raw material and the assembly of their functional composites with COFs. The strategy is to create new non-noble metal plasmonic/semiconducting hybrid materials based on strong electrostatic interaction in the water, particularly by hybridizing opposite-charged CuFeS_2_ nanoparticles with COF. New optical aspects arose in the hybrid assemblies, including the shift of absorbance by the plasmonic oscillations and the photoluminescence quenching. An understanding of the transitions influenced by the incorporation of the nanoparticles is realized through time-resolved fluorescence and Stern–Volmer analysis plots. The evaluation of the energy levels demonstrated a combination of electron and energy transfer pathways that elucidated the quenching mechanism of the frameworks.

## Experimental

### Synthesis of organophilic CuFeS_2_ nanoparticles

The CuFeS_2_ nanoparticles were synthesized following our previously reported methodology based on the utilization of elemental sulfur-amine solutions for the synthesis of metal sulfides colloidal particles.^28^ In a typical experimental procedure equimolecular Cu^2+^ and Fe^3+^ amounts in the form of metal acetylacetonate salts dissolved in well-degassed oleylamine (>90% primary amine content), at 100 °C. The mixture remained under a continuous flow nitrogen blanket, followed by the injection of elemental sulfur-oleylamine solution, with 10% excess in sulfur. Then, the temperature was raised to 250 °C and remained at this temperature for 1 h. Finally, the solution was cooled to room temperature and the formed nanoparticles were precipitated by the addition of ethanol and separated by centrifugation. The process was repeated several times to ensure the removal of any reaction byproducts and non-bonded amine molecules. The organophilic CuFeS_2_ nanoparticles were dissolved and stored in a *n*-hexane solution and they are denoted as CuFeS_2_-OP.

### Rendering the CuFeS_2_ nanoparticles hydrophilic

The hydrophobic oleylamine-capped CuFeS_2_ nanoparticles were converted to hydrophilic by a simple, previously reported procedure,^29^ which is based on the hydrophobic interactions between the aliphatic carbon chains of oleylamine and SDS molecules. Briefly, the solution of 15 g L^−1^ CuFeS_2_ nanoparticles in hexane was mixed with 10 ml of 5% w/v aqueous solution of sodium dodecyl sulfate, ((C_12_H_2_)_5_OSO_3_Na) (SDS), and sonicated until a homogeneous emulsion was obtained. Following, the emulsion was gently heated to 40 °C under magnetic stirring to completely remove the C_6_H_14_ phase. Any SDS excess was removed afterwards by dialysis using SnakeSkin 3500 membranes for 24 hours. The sample is denoted as CuFeS_2_–SDS and in the end, an aqueous solution with a concentration of 1 g L^−1^ CuFeS_2_–SDS was formed by diluting the initial stock solution. A density of CuFeS_2_ of 4.19 g cm^−3^ and an average radius of 5 nm is taken into consideration for the calculation of the molar ratio of the nanoparticles in the solution.

### Formation of hybrid COF@CuFeS_2_

The synthesis and exfoliation of the covalent organic framework have been published by Bika *et al.*^30^ The bulk material is denoted as bCOF and the exfoliated as exfCOF. For the formation of the hybrid materials, the first step was to disperse the COFs in H_2_O at a concentration of 0.2 g L^−1^ and subsequently, an appropriate quantity of nanoparticles was introduced. The hybrid mixtures were ultrasonically sonicated for 10 seconds at room temperature and depending on the characterization technique, the hybrid materials were either kept in their dispersion or as a solid powder after the evaporation of its medium. The new hybrid assemblies are denoted as bCOF@CuFeS_2_–SDS and exfCOF@CuFeS_2_–SDS.

### Characterisation techniques

Fourier transform infrared (FTIR) spectra for the solid samples were measured on a Thermo Nicolet iS50 instrument in attenuated total reflection mode from 400 cm^−1^ to 4000 cm^−1^. X-ray diffraction (XRD) patterns were completed in the 2*θ* range of 2–80° with a Siemens D500 X-ray diffractometer, using Cu-K_α_ radiation (*λ* = 1.5418 Å). Scanning electron microscopy (SEM) and FEI inspect microscope equipped with tungsten filament operating at 25 kV was used to investigate the morphology of the semiconducting powders, which were beforehand sputter-coated with Au. The FEI Talos F200i field-emission (scanning) transmission electron microscope (Thermo Fisher Scientific Inc., Waltham, MA, USA) was operating at 200 kV and it is equipped with a windowless energy-dispersive spectroscopy microanalyzer (6T/100 Bruker, Hamburg, Germany). The dispersions of the pristine and composite materials were deposited on copper grids for the TEM analysis. At the aqueous solutions and dispersions, zeta potential measurements were performed on a Malvern Instruments Zetasizer Nano™ Series analyzer. As the evaluation of the refractive index of the COFs and the nanoparticles was not possible, the software provided estimated data for similar materials: gold nanoparticles for CuFeS_2_ and polystyrene for the COFs. UV-visible absorbance spectra were carried out on an Analytic Jena Specord 210plus spectrophotometer with quartz cuvettes. Steady excitation-dependent photoluminescence (PL) spectra and maps were obtained on a JASCO FP-8300 spectrofluorometer. For the time-resolved fluorescence spectra, a time-correlated-single-photon-counting (TCSPC) method *via* a Horiba Fluoromax+ spectrofluorometer, a Delta-hub laser diode as an excitation source (404 nm, pulse duration < 150 ps) and the PMT detector were applied, along with a 450 nm optical filter intervened between the sample and the PMT. Ultraviolet photoelectron spectroscopy (UPS) measurements were carried out using a He excitation source with He I radiation at 21.22 eV and the spectra were collected with a PHOIBOS 100 (SPECS) hemispherical analyzer.

## Results and discussion

**Fig. 1:**
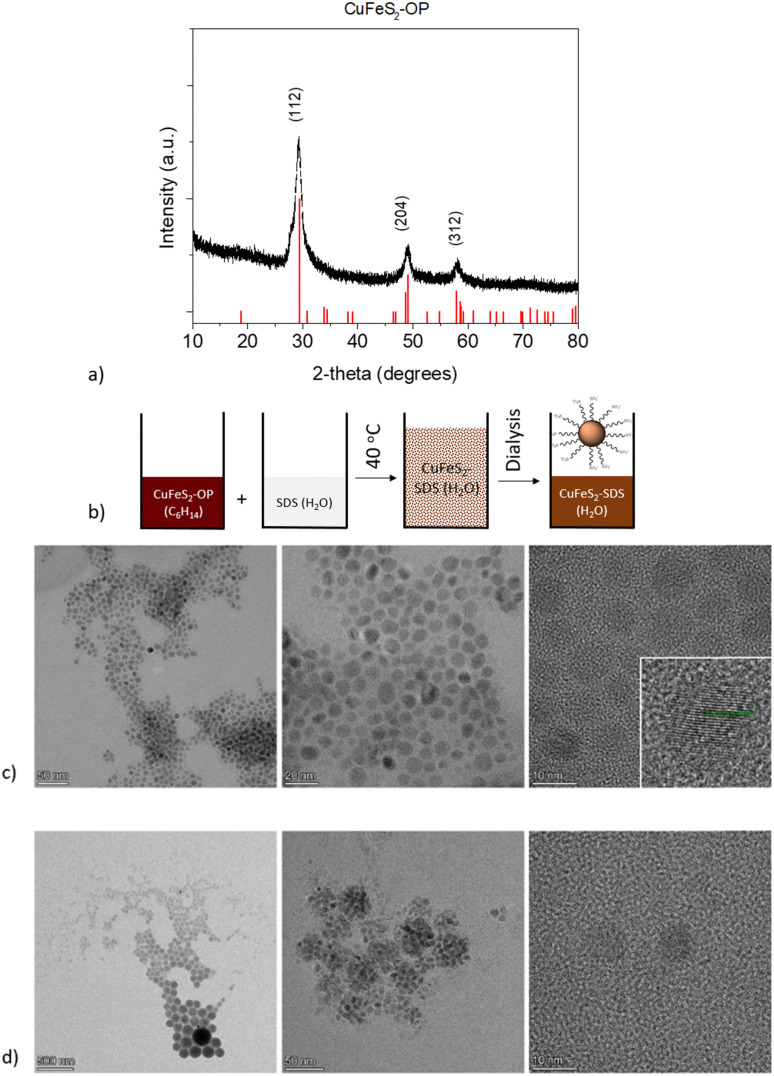
(a) XRD pattern of the as made CuFeS_2_-OP nanoparticles. (b) Schematic representation of the solubilization process of CuFeS_2_-OP to CuFeS_2_–SDS (c) TEM images of the organophilic nanoparticles dispersed in hexane (CuFeS_2_-OP), along with the HRTEM of the nanoparticles’ planes and (d) the hydrophilic functionalized nanoparticles dispersed in water (CuFeS_2_–SDS).

In the first step, organophilic CuFeS_2_ nanoparticles were synthesized utilizing sulfur waste and adopting an innovative approach, compared to the literature.^31^ The XRD pattern of the CuFeS_2_-OP nanocrystals shows intense diffraction peaks at 29.4, 49.1, and 58.3 2*θ*, which corresponds to the (112), (204), and (312) crystal planes, respectively. The pattern (Fig. 1a) confirms the formation of the tetragonal CuFeS_2_ crystal structure (JCPDS file no. 83-0983).

The second step involved the appropriate surface functionalization with anionic surfactants to render the particles hydrophilic, as depicted in Fig. 1b. The hydrophobic CuFeS_2_-OP nanoparticles were directed to the polar aqueous media through the water solubilization process, as described in the experimental section and as seen by the peaks of the surfactant at the lower degrees of the CuFeS_2_–SDS XRD pattern (Fig. S1, ESI†). The morphology and size of CuFeS_2_ were initially revealed with transmission electron microscopy and the images before and after the SDS functionalization, are presented in Fig. 1c and d. The as-made nanoparticles are monodispersed, uniform and have a diameter below 10 nm. Their crystalline nature, determined by HRTEM (Fig. 1c), shows the lattice fringes separated by the *d*-spacing calculated by fast Fourier transform (FFT) at 0.306 nm, which matches the spacing distance of the (112) plane. After their functionalization with SDS, the CuFeS_2_–SDS formed certain agglomerates, most likely induced by the interaction of the SDS chains.

The FTIR spectra of the nanoparticles, presented in Fig. S2 (ESI†), demonstrate the surface functionalization of the as-synthesized CuFeS_2_–OP and the CuFeS_2_–SDS. The characteristic bands of oleylamine are the in-plane CH_3_ terminal and CH_2_ stretching, which appeared at 2922 cm^−1^ and 2852 cm^−1^ respectively, whereas the vibrations of N–H located at 3322 cm^−1^ and 1929 cm^−1^, and C–C at 1652 cm^−1^ are absent. The successful exchange was confirmed by showing the elimination of the oleylamine features and the new vibrations of the CuFeS_2_–SDS nanoparticles, such as the –SO_2_ asymmetric stretching mode at 1220 cm^−1^ and the peaks at 3079, 2943, 2918, 2849 cm^−1^ of *v*(–C–H) stretching and bending modes of the surfactant,^32^ along with the presence of H_2_O at 3457 cm^−1^.

The optical properties of CuFeS_2_ vary depending on the chosen synthetic approach, alongside their chemical composition, precursor molecules, ligands, and diameter of the particles.^4^ Bulk chalcopyrite semiconductors exhibit an indirect band gap of 0.5–0.6 eV that can become broader due to strong quantum confinement effects by decreasing the nanoparticles’ size. Moreover, the overall band structure is consisted of valence (mainly Cu d orbitals and S p orbitals), intermediate state (a large amount of Fe d orbitals character) and conduction (almost equal contributors Cu, Fe, S) bands with electronic transitions from VB–IB (photobleaching) and IB–CB (induced absorption signal) as detailed in ref. 5, 33, 34 and 35.

**Fig. 2:**
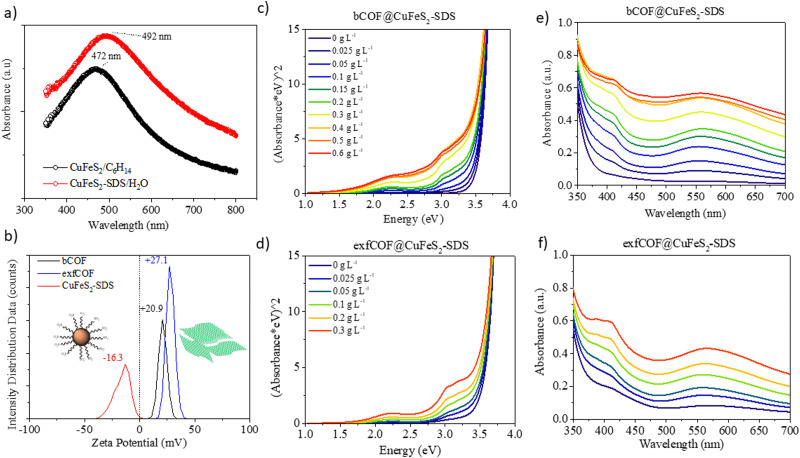
(a) UV-vis absorption of CuFeS_2_-OP and CuFeS_2_–SDS. (b) Zeta potential of the bCOF, exfCOF and the CuFeS_2_–SDS in water. (c) and (d) The Tauc plots of hybrid materials, depicting the increased absorption in the visible light range after the incorporation by increasing weight of the plasmonic nanoparticles in 0.2 g L^−1^ bCOF and 0.2 g L^−1^ exfCOF and (e) and (f) their corresponding UV-visible spectra.

The local surface plasmon resonance (LSPR) is developed when the size of the nanoparticles is smaller than the incident wavelength. The electron clouds oscillate harmonically, with the electromagnetic field and against the coulombic force evoked from the nucleus. The LSPR of the nanocrystals is sensitive to the type of the environment and their resonance conditions can be tuned or quenched depending on their dielectric properties.^6,36^ Moreover, the structural transformations in their stoichiometry could be accompanied by dramatic changes in the plasmonic peak maximum.^36^ In this regard, the UV-vis absorption spectra of the CuFeS_2_-OP in hexane and the CuFeS_2_–SDS in an aqueous medium are shown in Fig. 2a. The CuFeS_2_-OP in hexane demonstrated its plasmon resonance centred at 472 nm.^36^ The neutral nature of the oleylamine does not affect the charge carrier density.^35^ However, after capping the nanoparticles with SDS, the band is slightly broadened and red-shifted to 492 nm, in combination with the collective properties of the final agglomerates. The absorbance intensity of CuFeS_2_–SDS is increased, owing to scattering.^6^

The band gap of the nanoparticles was determined from the plot of (*Ahv*)^1/(1/2)^ (with *h* Planck's constant and *v* frequency) as a function of the photon energy (*E* = *hv*/*λ*) by extrapolating the slope from the edges to zero.^34^ The optical bandgap of CuFeS_2_-OP nanoparticles was evaluated at 2.03 eV (Fig. S3a, ESI†), a value larger than the bulk chalcopyrite, due to the size confinement effect of nanoparticles, as referred before. The band gap of the synthesized CuFeS_2_-OP nanoparticles is comparable and closely matches with previous results in the literature.^5–7,9,33,36–38^ When the nanoparticles were functionalized with SDS, the band gap decreased to 1.8 eV, due to the change of their dielectric environment. Additionally, according to the photoluminescence mapping, the CuFeS_2_-OP and CuFeS_2_–SDS nanoparticles are non-emissive in both dispersions (Fig. S3b, ESI†). It is proposed that the excited surface plasmons relax through non-radiative decay since the intermediate energy bands generate hot electrons, holes and heat.^7,39,40^

*Via* dynamic light scattering (DLS) and zeta potential, (Fig. 2b), the negative charge of the nanoparticles in H_2_O is verified at −16.3 mV, in contrast to the positive charges of bulk COFs at +20.3 mV and exfoliated COFs at +27.1 mV. The nanoparticles were functionalized with a negatively charged surfactant, namely sodium dodecylsulfonate derived from the terminal SO_3_^−^ group. Contrary, the COFs are positively charged due to the quaternized nitrogen. Generating attractive electrostatic interactions between opposed charged entities ensure new static and dynamic assemblies. The titration of the negatively charged inorganic constituent to the positively charged dispersion of the organic network assemblies the hybrid structures, only by changing the ionic environment, without any further modification of each system. The CuFeS_2_–SDS nanoparticles were kept stable in the aqueous solutions and a measured quantity was added to the dispersions of bulk and exfoliated COFs in H_2_O to create the hybrid materials in dispersions of different weight ratios. For further solid-state characterization, after 10 seconds of ultrasound sonication at the mixed dispersions, the water was left to evaporate at room temperature to obtain the hybrid powders.

To gain insight into the optical properties of the hybrid systems and to define the influence of the plasmonic nanoparticles on the absorption and fluorescence of the COFs and in the end, the electron or energy transfer between plasmonic and non-plasmonic components,^41^ the titration was followed by recording the UV-vis absorption. In Fig. 2c–f, the Tauc plots and the corresponding Uv-Vis spectra of both bulk and exfoliated hybrids are presented. The titration continued until the point where there was an excessive mass of nanoparticles in the dispersions. The positions of localized surface plasmon resonance at *λ* = 492 nm and COFs maximum absorption in the visible at *λ* = 550 nm were red-shifted to longer wavelengths (*λ* = 558 nm), due to the creation of hybrid assemblies and the dramatic change on the dielectric environment. It is perceived that an evolving plateau dependent on the weight ratio of nanoparticles is formed between 450 nm and 500 nm, that verifies the preservation of the SPR overlaid by COFs’ absorption at the newly developed static and dynamic superstructures even when the opposed charges of the materials are neutralized.^42^ The optical properties of the COF semiconductors are developed by electron delocalization and the lone pairs of nitrogen. The synergetic effect at the n–π* transitions becomes more evident at higher concentrations of nanoparticles in the dispersions, as the absorption of the hybrid materials is enhanced in the visible. The surface plasmon oscillations of the CuFeS_2_–SDS efficiently alter the optical properties of the semiconductor substrates at the near field, increasing the absorption of the 3D and 2D COF configurations and likewise, the electric field of the nanoparticles is influenced by the presence of COFs. Modifications are also observed further from the LSPR and specifically, in the UV range. At 400–410 nm, the absorption in both bCOF@CuFeS_2_–SDS and exfCOF@CuFeS_2_–SDS is enhanced, indicating an increase of the π–π* transitions of the reduced COF state and thus, an electron density redistribution in the hybrid system with the functionalized nanoparticles. The plasmonic oscillations affect the electron delocalization of the COFs and enhance their absorption, specifically in the visible range. The band gap of the hybrid materials is red-shifted (Fig. S4, ESI†), becoming narrower by increasing the concentration of the nanoparticles, indicating the generation of more photoinduced electron–hole pairs.^43,44^

In Fig. S4 (ESI†), the XRD patterns of the pristine COFs and hybrid materials are compared. The main characteristic peak at 27–28° (3.03–3.3 Å) of both layered COFs unveils their π–π stacking conjugation.^30^ In both hybrid materials, the intensity of the interlayer distance reflection is tremendously diminished due to the incorporation of the nanoparticles, signifying the complete formation of a hybrid material throughout the organic framework. Furthermore, new sharp peaks arise from the intercalation of the nanoparticles’ surfactant. The frameworks extend their pores to incorporate the CuFeS_2_–SDS, showing the *d*-spacing of SDS at 35 Å for the bCOF and 38 Å for the exfCOF. At both hybrid samples, the peaks of 4.5° (19 Å) and 6.7° (13 Å), attributed to the presence of SDS, are slightly distinguishable.

FTIR reveals additional peaks that originate from the incorporation of CuFeS_2_–SDS inside the COFs at the range of 2700–2900 cm^−1^. These peaks are assigned to the surfactant and are demonstrated by red arrows in Fig. S5 (ESI†).

**Fig. 3:**
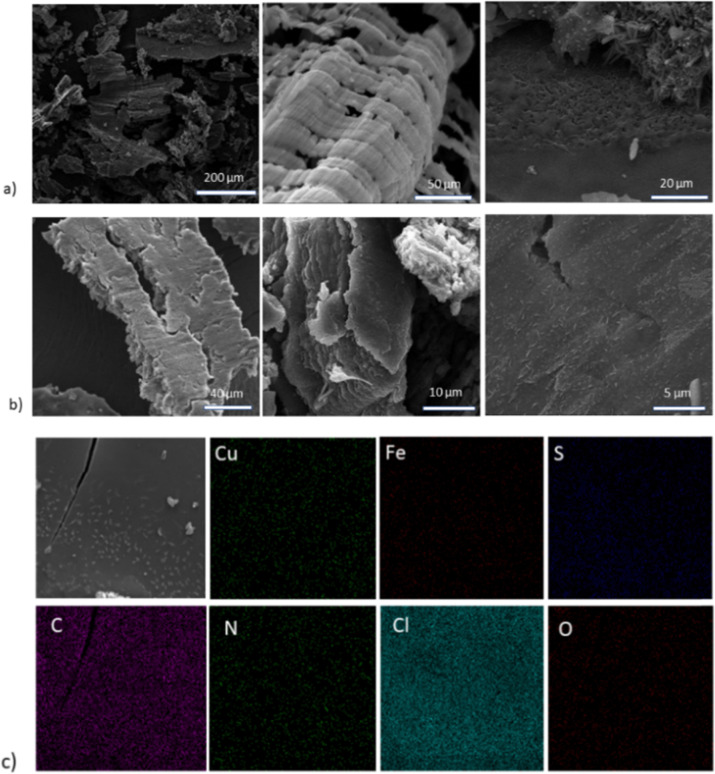
SEM images of the (1–1) (a) bCOF@CuFeS_2_–SDS and (b) exfCOF@CuFeS_2_–SDS hybrid materials. (c) EDX mapping of the Cu, Fe, and S elements of the nanoparticles on bCOF@CuFeS_2_–SDS.

SEM images were obtained to identify the morphology of the hybrid materials, presented in Fig. 3, alongside the corresponding EDX mapping. Upon the addition of CuFeS_2_–SDS nanoparticles, the amorphous bCOF is transformed into expanded layered sheets with length at the micrometre scale. The nanoparticles of CuFeS_2_–SDS were not observable with SEM owing to their small size. Instead, the elemental mapping of the hybrid bCOF@CuFeS_2_–SDS certifies the presence of nanoparticles and a homogeneous distribution of Cu, Fe, and S elements on the surface of the hybrid material. At this step, the energy dispersive X-ray analysis first confirms the presence of the nanoparticles on the bulk hybrid (Table S1 and Fig. S6, ESI†) and then, qualitatively estimates the composition of the nanoparticles, which have an atomic ratio close to the theoretical stoichiometry. The functionalized nanoparticles are diffused through the pores and are positioned between the layers of the bCOF, provoking its partial exfoliation based on Fig. 3a. This is also supported by the decrease of the π–π stacking peak at the XRD pattern. Moreover, at the exfCOF@CuFeS_2_–SDS sample, the microscopy images demonstrate the formation of arranged blocks (Fig. 3b). The configuration of the hybrid layers seems to be epitaxial.

**Fig. 4:**
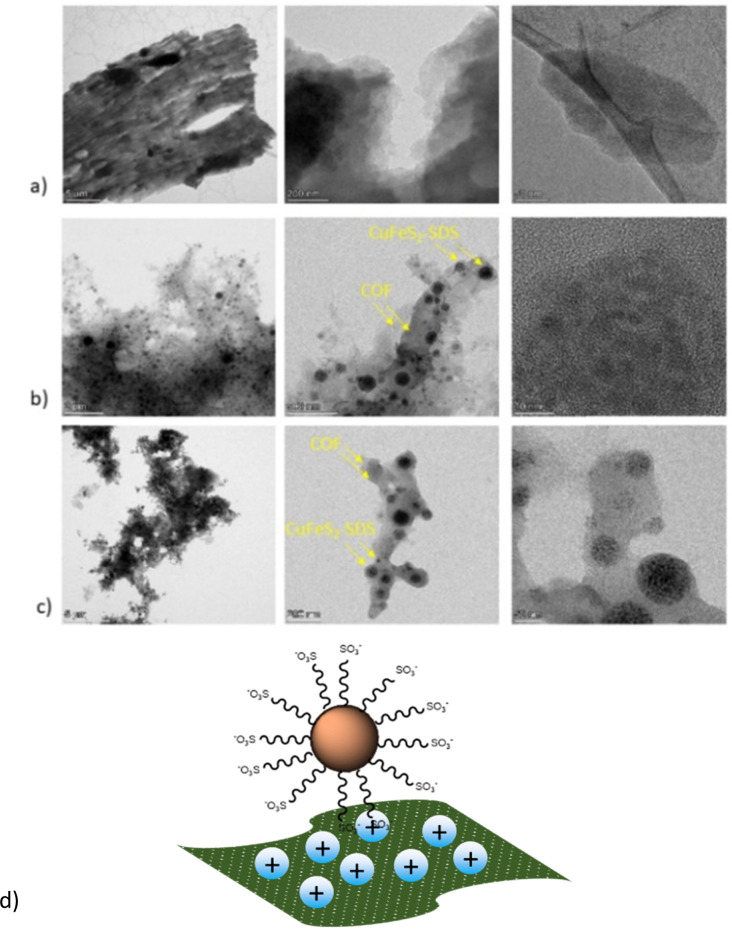
TEM images of (a) bCOF, (b) (1–1) bCOF@CuFeS_2_–SDS and (c) (1–1) exfCOF@CuFeS_2_–SDS (d) and a schematic representation of the as-formed hybrids.

The TEM images are presented in Fig. 4 to further analyze the morphology of pristine and hybrid systems. Remarkably, all nanoparticles were immobilized on the COFs as the depositions of the hybrid dispersions on the grid had shown, making evident their strong electrostatic interactions and the dense surface loading through the bottom-up route. Additionally, both COFs could have also provided numerous nitrogen active sites for the nanoparticles to anchor. In the end, the main sheet morphology of the networks was not altered, as seen from the nanometer-scaled images in Fig. 4a and b. A schematic representation of the hybrids structure can be also found in Fig. 4d.

**Fig. 5:**
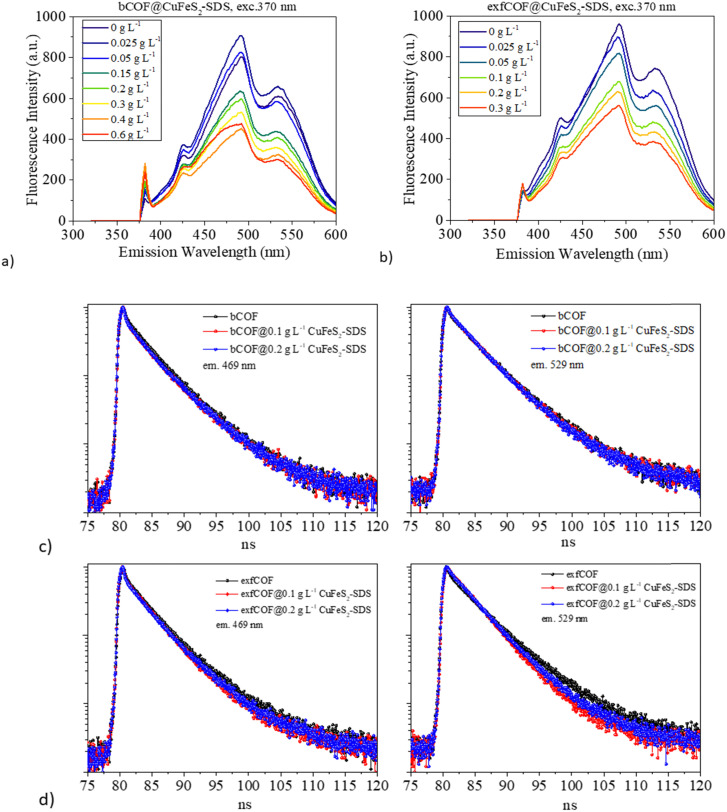
(a) and (b) Steady-state photoluminescence of the 0.2 g L^−1^ bCOF and 0.2 g L^−1^ exfCOF and their hybrid materials. (c) Time-resolved fluorescence for 0.2 g L^−1^ bCOF, bCOF@CuFeS_2_–SDS and (d) 0.2 g L^−1^ exfCOF, exfCOF@CuFeS_2_–SDS after the addition of 0.1 and 0.2 g L^−1^ CuFeS_2_–SDS at *λ*_em_ = 469 & 529 nm.

Excitation-dependent photoluminescence mapping (Fig. S7, ESI†) was employed to identify the optimum excitation wavelength and to further understand the impact of plasmonic nanoparticles on the optical properties of COFs. The emission of both pristine and hybrid assemblies was then studied by recoding the steady-state photoluminescence spectra with the optimum excitation at 370 nm (Fig. 5a and b). In general, the exfoliated, 2D materials hold promising aspects compared to bulk materials, such as higher carrier mobility and electron transfer, larger available surface areas and even the presence of defects, dopants or oxygenated functional groups are beneficial for the enhancement of their optical response by the accumulation of plasmonic nanoparticles on their substrates.^45,46^ Systems of 2D materials have carriers with shorter diffusion lengths and easier migration to the surface.^47^ This is the reason why before the hybrid assembly and while their emission states are the same, the pristine exfCOF has a higher fluorescence and, thus higher radiative recombination than the pristine bCOF, in accordance with previous work.^30^

Since the CuFeS_2_–SDS nanoparticles dispersed in H_2_O are non-emissive (Fig. S3, ESI†), their surface plasmons relax through non-radiative damping, owing to their intermediate energy gaps. They combine the formation of electron–hole pairs by intra and inter-band excitations^48^ and the photothermal conversion.^7^ These light-absorbing nanoparticles can transfer the plasmon energy to their surrounding,^49^ preferably by hot electron injection.^1,50^

The collective electromagnetic oscillations on the hybrid assemblies are affecting the photoluminescence of the COFs. In Fig. 5a and b, it is evident that the photoluminescence of the COFs was quenched with the addition of the plasmonic nanoparticles, suggesting the reduced radiative recombination of e^−^–h^+^ pairs, whereas no energetic shifts of the emission peaks are observed. The electron transfer takes place from the COFs to the CuFeS_2_–SDS nanoparticles interface^43,51,52^ with the nanoparticles acting as an electron reservoir. Nevertheless, there are additional reasons for the PL quenching near metallic nanoparticles.^53^ In both hybrid materials, the increase of the filling factor nanoparticles’ population on the COFs means a high coverage that concentrates and absorbs much of the incident light. The SPR absorption (based on the plasmon radiating model of Lakowitz^53^) reduces the PL response of the semiconductor.^54,55^ Moreover, a major factor influencing the PL intensity is the inter-distance between the two components. When the distance is shorter than the optimal gap of 10 nm,^16,56^ the PL quenching is induced, as the plasmonic oscillations are stacked.

Afterwards, the excitation wavelength was set at 404 nm, in distance to the LSPR of the CuFeS_2_–SDS nanoparticles. This excludes the excitation enhancement parameter in the fluorescence and lifetime response. Correspondingly, the time-resolved fluorescence spectra of the pristine and hybrid materials were recorded under 404 nm pulsed laser excitation at the emissions of 469 nm and 529 nm (Fig. 5c and d). The decay curves were fitted with a bi-exponential function and the lifetimes of the radiative and non-radiative carriers with their fractional amplitudes are gathered in Fig. S8 (ESI†). The values of the radiative lifetime are gathered in Table 1, where *τ*_0_ is the lifetime of the pristine materials without the addition of the nanoparticles.

**Table 1:** The radiative lifetimes of the pristine and hybrid materials are summarized

Sample	*τ*_rad_ (469 nm)	*τ*_rad_ (529 nm)	*τ*_0_/*τ* (469 nm)	*τ*_0_/*τ* (529 nm)
bCOF	4.9	4.7	1.0	1.0
bCOF@0.1 g L^−1^ CuFeS_2_–SDS	5.3	4.4	0.9	1.1
bCOF@0.2 g L^−1^ CuFeS_2_–SDS	5.3	4.4	0.9	1.1
exfCOF	4.1	4.7	1.0	1.0
exfCOF@0.1 g L^−1^ CuFeS_2_–SDS	4.1	3.9	1.0	1.2
exfCOF@0.2 g L^−1^ CuFeS_2_–SDS	5.0	3.9	0.8	1.2

Along with the PL quenching of the plasmonic-based hybrid materials and thus the decrease of the radiative recombination or increase of the non-radiative rate, there are alternations provoked at the lifetimes of the carriers that are dependent on the concentration of the nanoparticles and the distance of the emission from the SPR. At 469 nm, there is a slight increase in the lifetimes, whereas at 529 nm, a reduction is observed with the addition of CuFeS_2_–SDS. The reduction of the carriers’ lifetime is a consequence of an effective separation of electron–hole pairs, a pronounced charge transfer interaction^51^ with faster diffusion at a donor–acceptor system and the creation of new non-radiative pathways.^57^ While the elongation of the lifetime suggests a higher probability for migration to the interface,^25,43^ and a longer pathway to the ground state thanks to the interaction of the two components, the accumulation of the charges to the interface and the electron delocalization from the SPR.

**Scheme 1:**
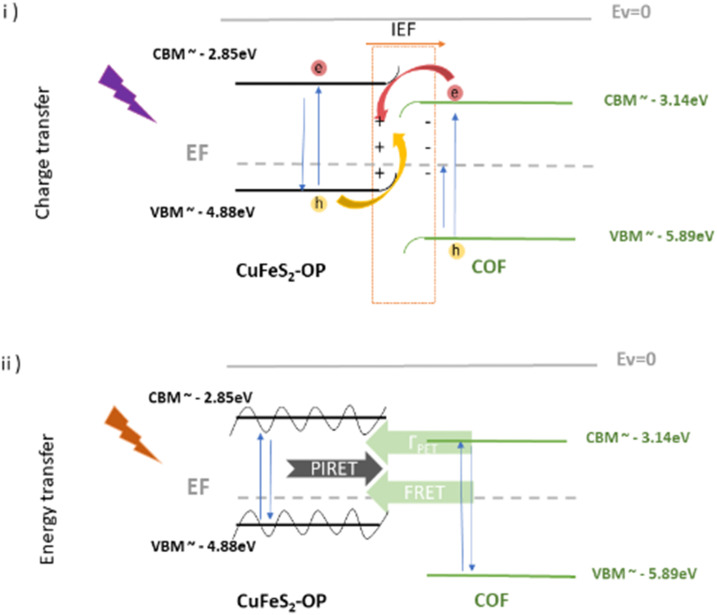
Schematic representation of the band energy positions of CuFeS_2_-OP nanoparticles and the COF, along with the proposed transfer mechanisms after contact and under (i) UV and (ii) visible illumination at the S-scheme.

The decreased recombination rate of the charge carriers and the lifetime responses can be explained by the energy or electron transfer mechanism between the two semiconducting materials. In order to have a better understanding of the electronic states of the nanoparticles and the COFs, along with the mechanistic pathways affecting the optical properties of the hybrids, UPS measurements were conducted to the CuFeS_2_-OP nanoparticles (Fig. S9, ESI†) and the COF electronic states were converted accordingly from NHE to vacuum. The band alignment is presented in Scheme 1, suggesting that the nanoparticles are p-type, considering their Fermi level and in agreement with,^33^ while the COFs are proven to be n-type.^21^ Once the hybrid system is formed and upon close contact, there would be a spontaneous electron transfer from the CuFeS_2_ to the COF in the interfacial connection, until Fermi alignment (Scheme 1i). In consequence, the COF would accumulate electrons, whereas the CuFeS_2_ would be depleted, leading to the creation of an internal electric field at their interface, leading to band edges bending and coulomb interactions. The electron transfer direction is then reversed upon UV illumination, and an S-scheme heterojunction electron–hole transport pathway is established.^58^ The photogenerated electrons of the COF in the CB are transferred to the VB of CuFeS_2_ through the contact interface, they are recombined with the holes and then they are excited to the CB of CuFeS_2_, inducing also a longer lifetime. Thus, the CuFeS_2_ is an electron reservoir, and the holes are concentrated in the VB of the COF. This S-scheme system is highly recommended for photocatalytic applications, leading to a spatial distribution of reduction and oxidation carriers.^59–62^

Furthermore, an additional mechanism is proposed if the excitation wavelength is close to the SPR at the visible range. When there is an overlap of the CuFeS_2_ absorption with the COFs’ emission, at the SPR wavelength, the energy exchange is likely to happen through plasmon–exciton interactions,^63^ including forward (PIRET) and backward (FRET) directions of energy flow, quenching the system and therefore inducing a faster lifetime^64–67^ (Scheme 1ii). The quenching of fluorescence can be attributed to the LSPR-mediated local heating of the surrounding environment, but it's excluded from this study, as also the potential influence of the temperature.^66^ The FRET formalism for the energy transfer efficiency in a donor–acceptor system is represented by *E* = 1 − *τ*/*τ*_0_.^64^ The equation is accurate, specifically when the energy transfer is the primary mechanism of the donor emission quenching (negligible charge transfer contribution). The percentages were calculated for the COF@CuFes_2_–SDS materials. Specifically, at *λ* = 529 nm, the energy transfer happens with a 7% at the bCOF and with 18% efficiency at the exfCOF, thanks to the overlapping of the nanoparticles’ absorbance and the COFs emission. Contrarily, at *λ* = 469 nm, there is only an electron transfer expected in the hybrid systems.

**Fig. 6:**
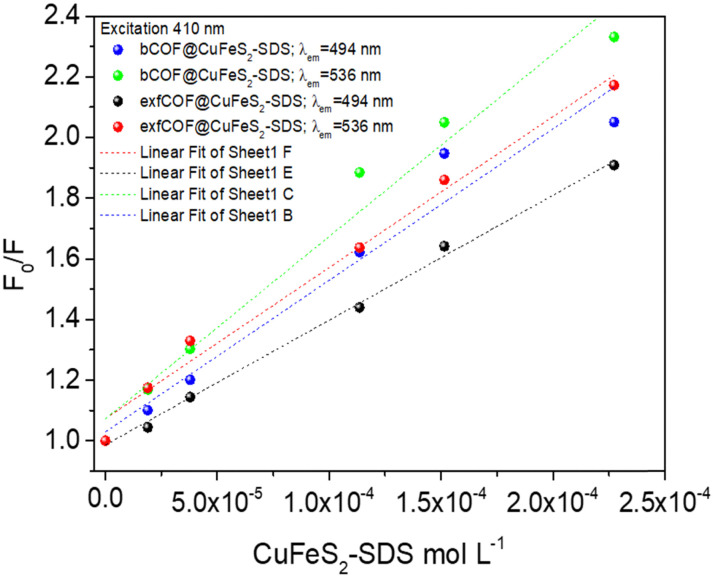
The Stern–Volmer (*F*_0_/*F*) plots at 494 nm and 536 nm emissions of bCOF@CuFeS_2_–SDS and exfCOF@CuFeS_2_–SDS under steady-state excitation of *λ*_exc_ = 410 nm.

To further examine the fluorescence quenching at the bCOF@CuFeS_2_–SDS and exfCOF@CuFeS_2_–SDS hybrid systems, we proceeded with the Stern–Volmer analysis. The fluorescence intensity ratio (*F*_0_/*F* = initial fluorescence without/with the quencher) was plotted in Fig. 6a dependent on the concentration of the quencher (*i.e.* CuFeS_2_–SDS).^68^ In most cases, the dynamic or collisional quenching represents the diffusive collisions between the fluorophore and quencher, during the lifetime of the excited state and it is governed by the linear S–V equation:^17^1
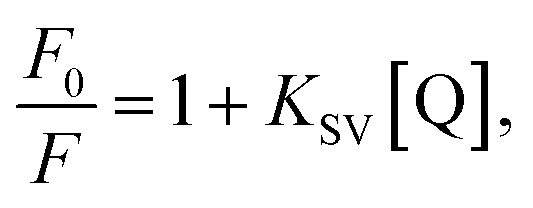
with *K*_SV_ = *k*_q_*τ*_0_, in the absence of quencher. In static quenching or else contact quenching, the quencher forms a non-fluorescence complex with the fluorophore in their ground state and its equation is represented by the extended S–V:2
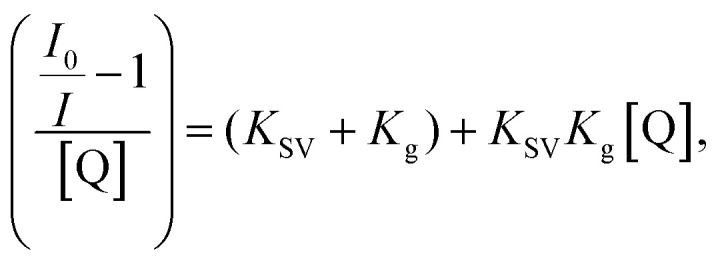
with *K*_g_ the complex association constant.

In Fig. 6, the S–V plots of bCOF@CuFeS_2_–SDS and exfCOF@CuFeS_2_–SDS, excited at 410 nm by steady-state photoluminescence were fitted by a linear equation and its parameters are presented in Fig. S10 (ESI†). The plots have a mostly linear response for both emissions, indicative of one quenching mechanism, and the *K*_SV_ and *k*_q_ constants are presented in Table 2. The bCOF@CuFeS_2_–SDS may deviate from linearity with the increase of the concentration (higher than 1.0 × 10^−4^ mol L^−1^), as the fitting error is beyond the acceptable limit (Fig. S10, ESI†) and a downward curve seems to be shaped (Fig. S11, ESI†), due to the presence of two populations of fluorophores (heterogeneous quenching), one of which is not accessible,^69^ possibly attributed to the influence of plasmonic oscillations or the self-aggregation of COFs’ dimeric species.^17^ Additionally, suggestions for the type of quenching can be given also by the results of UV-vis spectroscopy, since shifts in absorption peaks and new absorption bands are characteristic of the static one.^17,70^

**Table 2:** *K*_SV_ constant derived from the linear S–V fitting and *k*_q_ (min, max) coefficients regarding *τ*_0_ of both hybrid systems at the 494 nm and 536 nm emission states

*λ*_exc_ = 410 nm	(M^−1^) *K*_SV_ at 494 nm	(10^9^ M^−1^ s^−1^) *k*_q_ at 494 nm	(M^−1^) *K*_SV_ at 536 nm	(10^9^ M^−1^ s^−1^) *k*_q_ at 536 nm
bCOF@CuFeS_2_–SDS	4996.32 ± 514.93	(914, 1127)	6011.70 ± 495.38	(1173, 1384)
exfCOF@CuFeS_2_–SDS	4116.48 ± 112.68	(976, 1031)	4979.13 ± 279.62	(999, 1118)

To further elucidate the quenching mechanism, the decay lifetimes of the hybrid materials were also taken into consideration. Based on the *τ*_0_/*τ* values (Table 1), in 0.1 g L^−1^, a dynamic quenching is pointed out as there is a change in the lifetime response of the excited state, whereas, at the 0.2 g L^−1^, a static quenching mechanism of photoluminescence is observed with no further changes, denoting the formation of non-fluorescent ground states. Since there are a lot of phenomena emerging from this system, a distinction between the contribution percentages of dynamic and static quenching is difficult.

Regardless, the coefficient rates of both hybrid systems in Table 2 calculated from eqn (1), demonstrate an efficient dynamic quenching and their values ensure the high proximity of COFs with the chalcogenide.^71,72^ Likewise, Fan *et al.*^73^ demonstrated that the strong electrostatic interaction between the plasmonic particle and the fluorophore is crucial for the energy transfer, using negatively charged gold and cationic conjugated polymers. However, in Fig. S11 (ESI†), based on the eqn (2), the extended S–V plot of the bCOF@CuFeS_2_–SDS permits a better fitting of the values (*R*^2^ = 0.99) than the linear S–V of eqn (1), (Fig. 6). By replacing the parameters and solving the 2nd order differential equation of *K*_g_^2^-intercept *K*_g_ + slope = 0, the results are +1914 M^−1^ or −7468 M^−1^ for 494 nm and +1280 M^−1^ or −10 234 M^−1^ for 536 nm. Between those two values for each occasion, the complex association constant *K*_g_ is the positive one. Therefore, since both *k*_q_ and *K*_g_ are comparable, at the bCOF@CuFeS_2_–SDS system, both static and dynamic quenching mechanisms are active. Interestingly, it is observed that the bCOF@CuFeS_2_–SDS presents enhanced amplification in comparison to the exfCOF@CuFeS_2_–SDS, since the increased degree of conjugation of the bCOF offers a superior probability of attachment, *via* stronger capture ability through electrostatic interaction,^74^ multiply exciton migration and rapid energy and electron transport pathways. For both hybrid systems, the slightly higher value of *k*_q_ on the 536 nm emission state than the one at 494 nm, suggests the interference of the plasmon oscillations, when there is an overlap of the absorption of the quencher with the emission of the fluorophore. The exciton–plasmonic weak coupling is reinforced as indicated previously, by electron transfer near the SPR^56,63^ increasing the quenching rate and providing a faster diffusion.

## Conclusions

A new facile route by utilizing elemental sulfur waste is presented for the synthesis of CuFeS_2_ nanoparticles, which were further incorporated into covalent organic frameworks. Their surface plasmon resonance is sensitive to the dielectric environment and differences are evoked with their transfer to the polar solvent and the subsequent formation of nanoparticle assemblies. By rendering the nanoparticles negatively charged, the electrostatic assembly with the positively charged COFs in water was achieved, without altering their sheet morphology, as evidenced by electron microscopy studies. The optical properties of the 3D and 2D COFs are altered with the addition of the plasmonic nanoparticles and Stern–Volmer analysis was employed for the evaluation of the quenching mechanism, with the bulk COF demonstrating a higher transfer rate compared to the exfoliated materials. Extensive steady-state and time-resolved fluorescence data revealed the inhibition of the radiative recombination, exciting the hybrid system on or far from the surface plasmon resonance. The effective electron transfer takes place from the COFs donors to the CuFeS_2_ acceptors following an S-scheme pathway and an increase of the carriers’ lifetime. The energy exchange occurs, only when there is an overlap of the nanoparticles’ absorption and COFs’ emission, initiating a faster diffusion. This study is of significant interest in the fields of photocatalysis and photovoltaics, as it reveals for the first time the strong interaction of COFs coupled with the CuFeS_2_ nanoparticles and their optical properties, on and off plasmon resonance.

## Data availability

Data for this article are either included in the manuscript and the supporting information or are available upon request.

## Author contributions

P. B.: conceptualization; data curation; formal analysis; investigation; methodology; validation; writing – original draft. V. T.: conceptualization; data curation; formal analysis; investigation; methodology; validation; visualization. E. S.: data curation; formal analysis; investigation. S. O.: data curation; formal analysis; investigation. N. B.: data curation; formal analysis; investigation. S. M. A.: conceptualization; resources. P. T.: data curation; formal analysis; investigation. V. P.: formal analysis; investigation. T. S.: funding acquisition; methodology; resources; validation. P. D.: conceptualization; data curation; funding acquisition; investigation; methodology; project administration; resources; supervision; validation; visualization; writing – original draft.

## Conflicts of interest

There are no conflicts to declare.

## Supplementary Material

TC-012-D4TC01989J-s001
