# Supplementary material for: Electron transfer and energy exchange between a covalent organic framework and CuFeS2 nanoparticles
Source: J Mater Chem C Mater. 2024 Jun 14;12(28):10475–86. doi: 10.1039/d4tc01989j (PMC11257035; doi:10.1039/d4tc01989j)
Supplement: TC-012-D4TC01989J-s001 [file TC-012-D4TC01989J-s001.pdf]

Electronic Supplementary Material (ESI) for Journal of Materials Chemistry C.
This journal is © The Royal Society of Chemistry 2024

Supporting Information

Electron transfer and energy exchange between a covalent organic framework and $CuFeS_2$ nanoparticles

Panagiota Bika[a], Vasileios K. Tzitzios[a], Elias Sakellis[a], Spyros Orfanoudakis[a,b], Nikos Boukos[a], Saeed M. Alhassan[c], Polychronis Tsipas[a,d], Vasileios Psycharis[a], Thomas Stergiopoulos[a*] and Panagiotis Dallas[a**]

[a] *Institute of Nanoscience and Nanotechnology, NCSR Demokritos, 15341, Athens, Greece.*

[b] *School of Applied Mathematical and Physical Sciences, National Technical University Athens, 15780 Zografou, Athens, Greece.*

[c]*Department of Chemical Engineering, Khalifa University of Science and Technology, P.O. Box 127788, Abu Dhabi, United Arab Emirates.*

[d] *National Institute of Materials Physics, Atomistilor 405A, Magurele, Romania.*

Keywords: fluorescence quenching, chalcogenides, covalent organic frameworks, plasmonic nanoparticles, Stern-Volmer analysis

* t.stergiopoulos@inn.demokritos.gr; Tel: +302106503394

** p.dallas@inn.demokritos.gr; Tel: +302106503311

Supporting Information

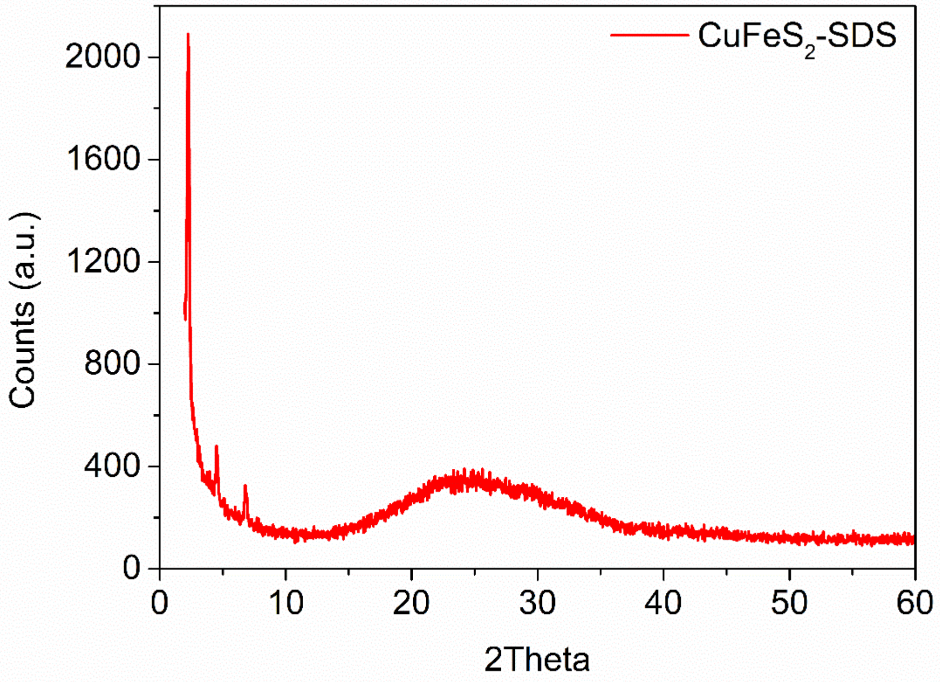

Figure S1. XRD pattern of the $CuFeS_2$-SDS nanoparticles on a glass substrate.

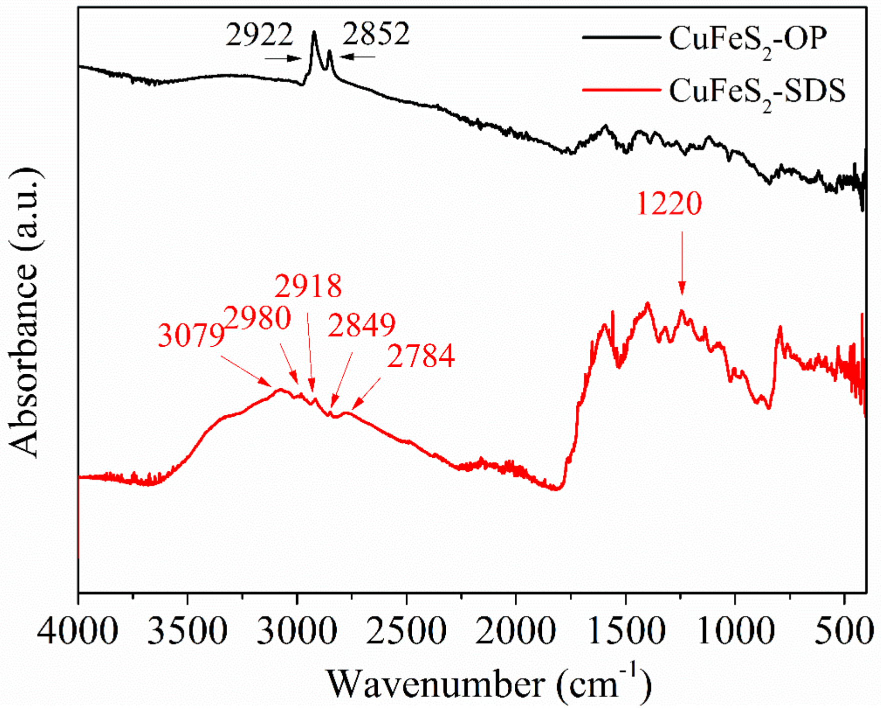

Figure S2. FTIR spectra of $CuFeS_2$-OP vs the hydrophilic $CuFeS_2$-SDS

Supporting Information

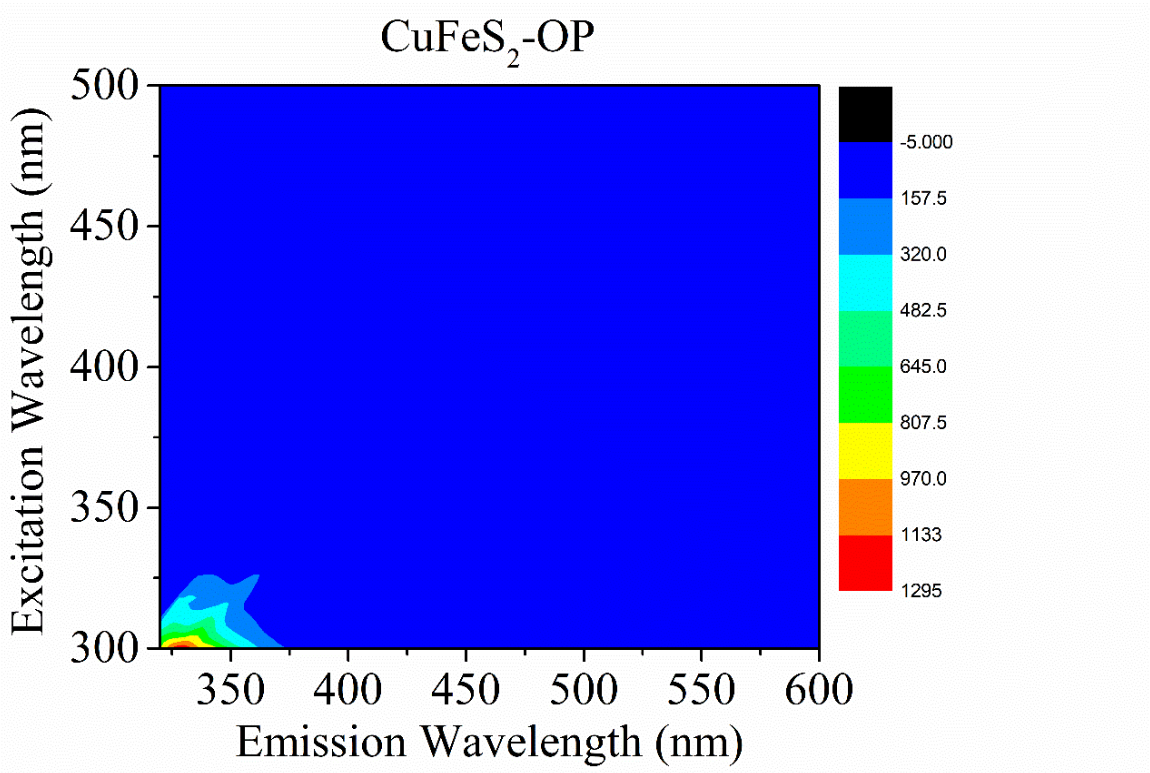

a)

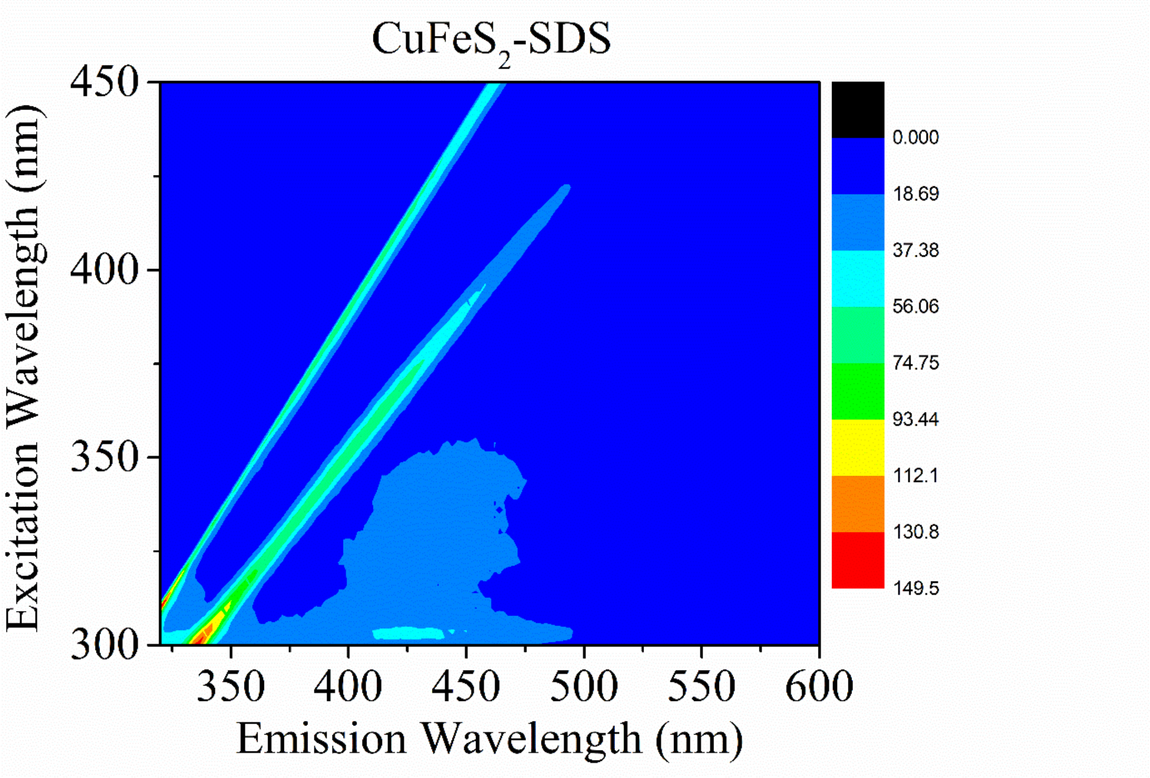

b)

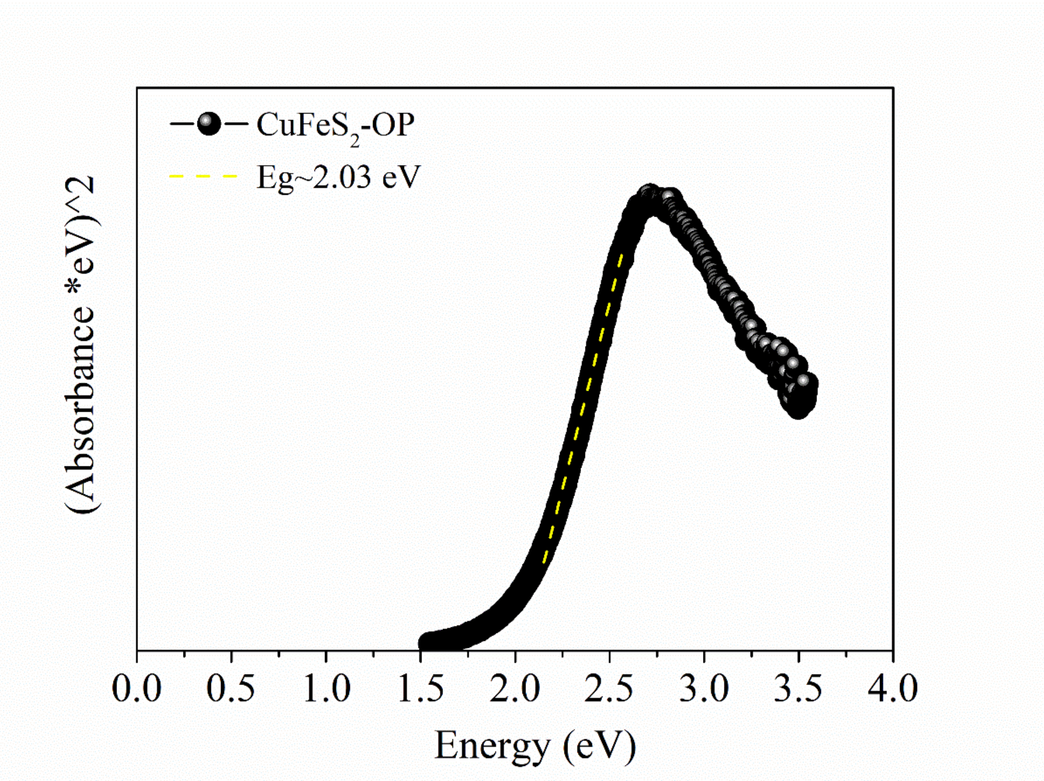

c)

Figure S3. Excitation dependent photoluminescence mapping for a) $CuFeS_2$-OP b) $CuFeS_2$-SDS c) Tauc plot for the $CuFeS_2$-OP considering a direct band gap

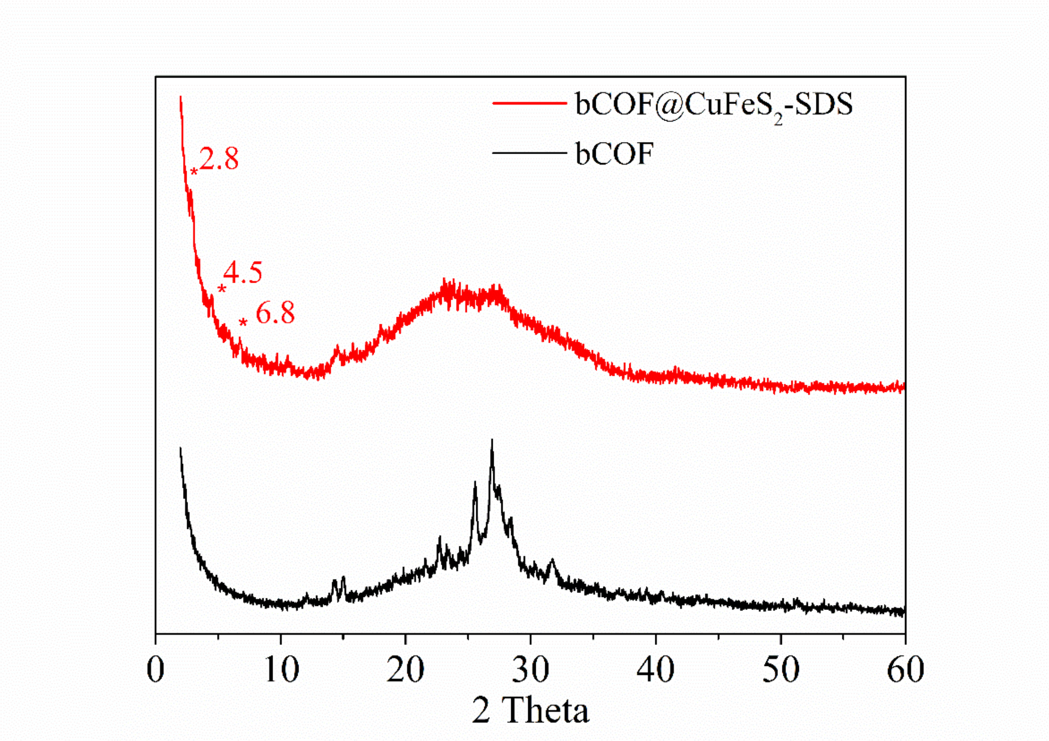

a)

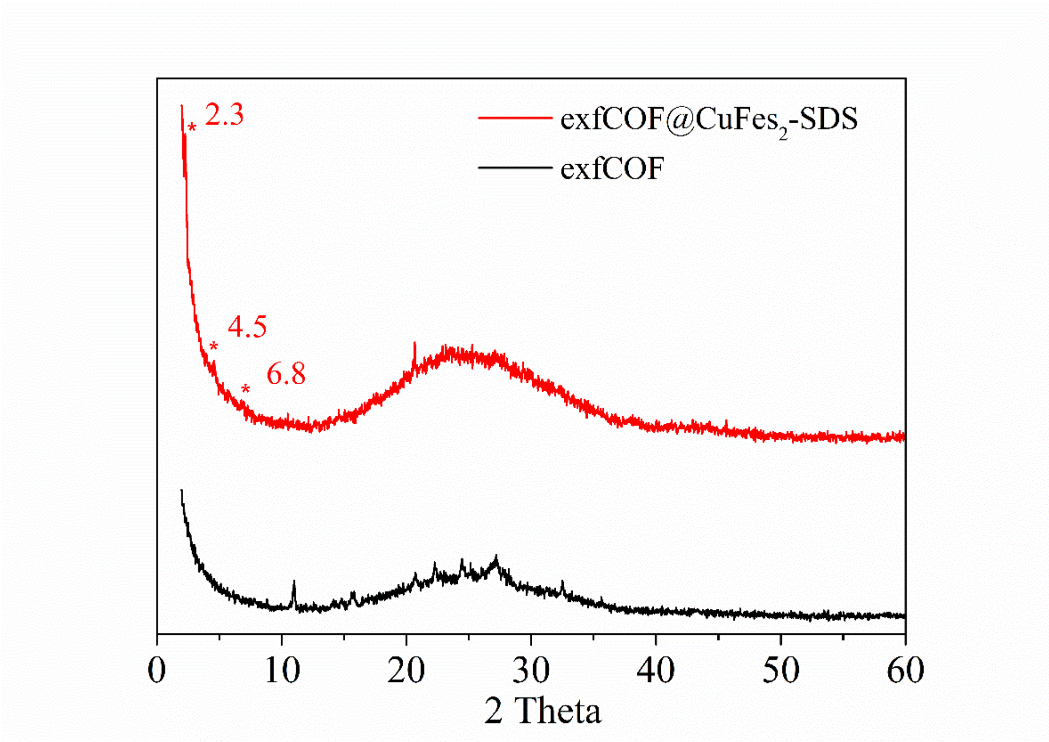

b)

Figure S4. XRD patterns as a comparison between the pristine bulk and exfoliated COFs vs their hybrid materials with $CuFeS_2$-SDS

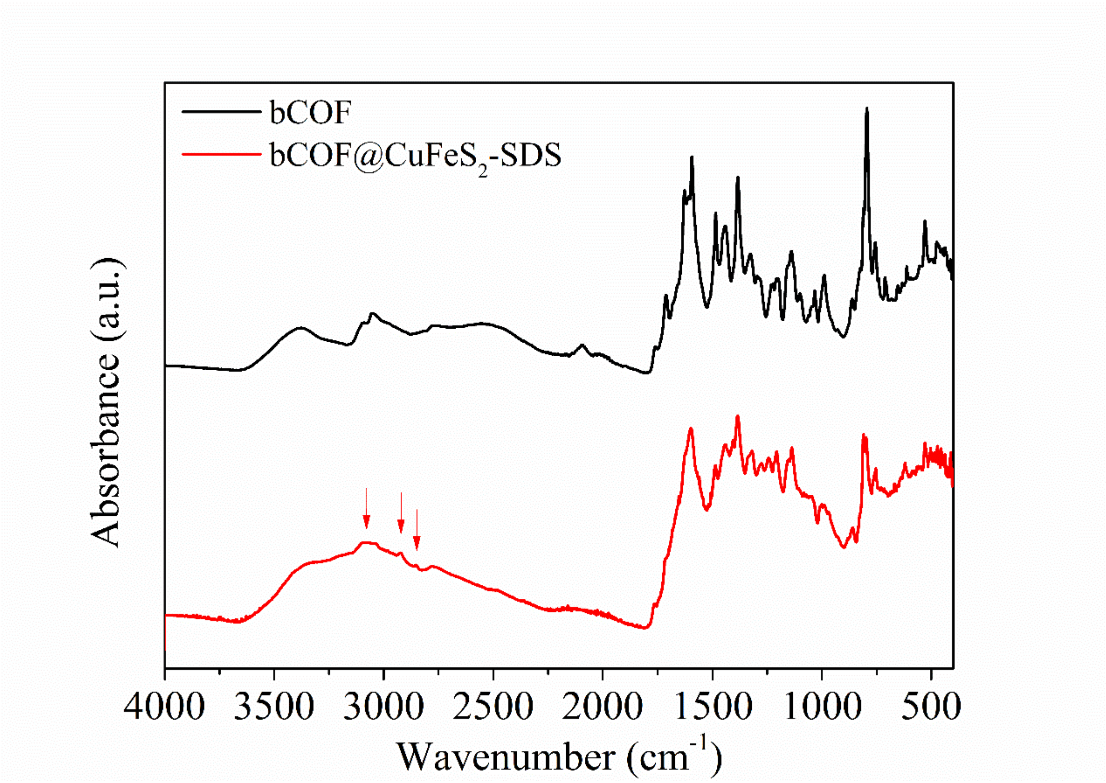

a)

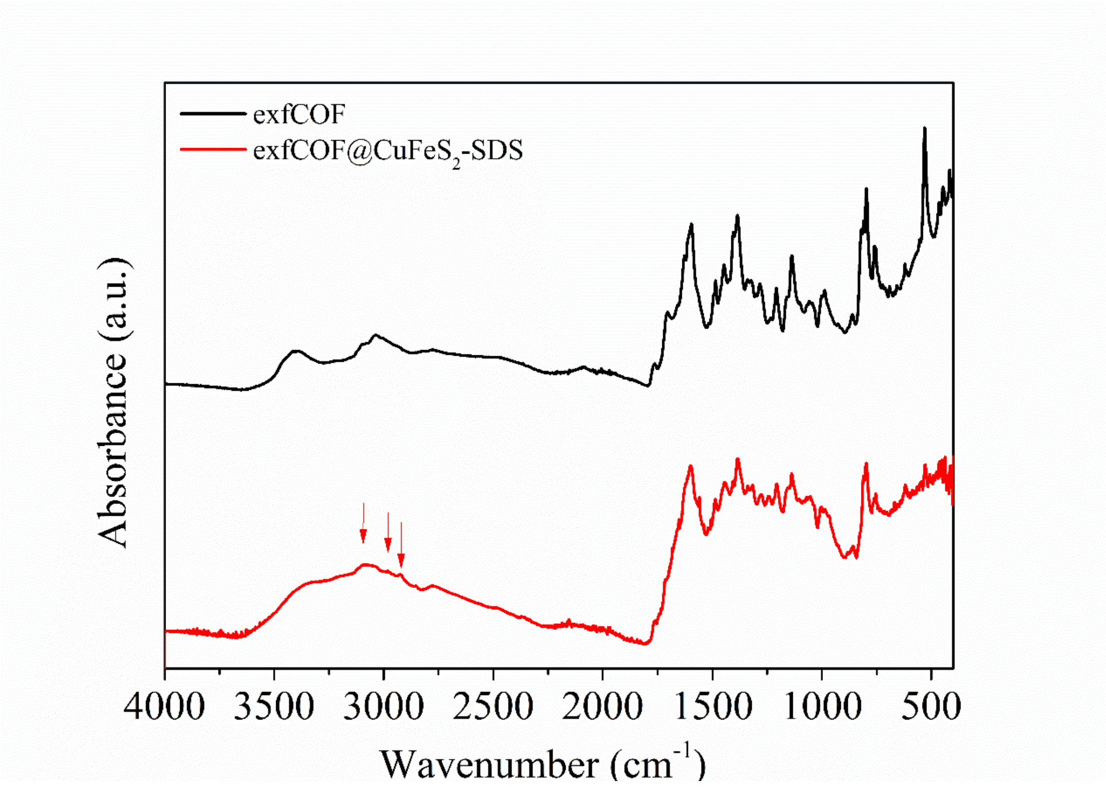

b)

Figure S5. FTIR spectra as a comparison between the pristine bulk and exfoliated COFs vs their hybrid materials with $CuFeS_2$-SDS

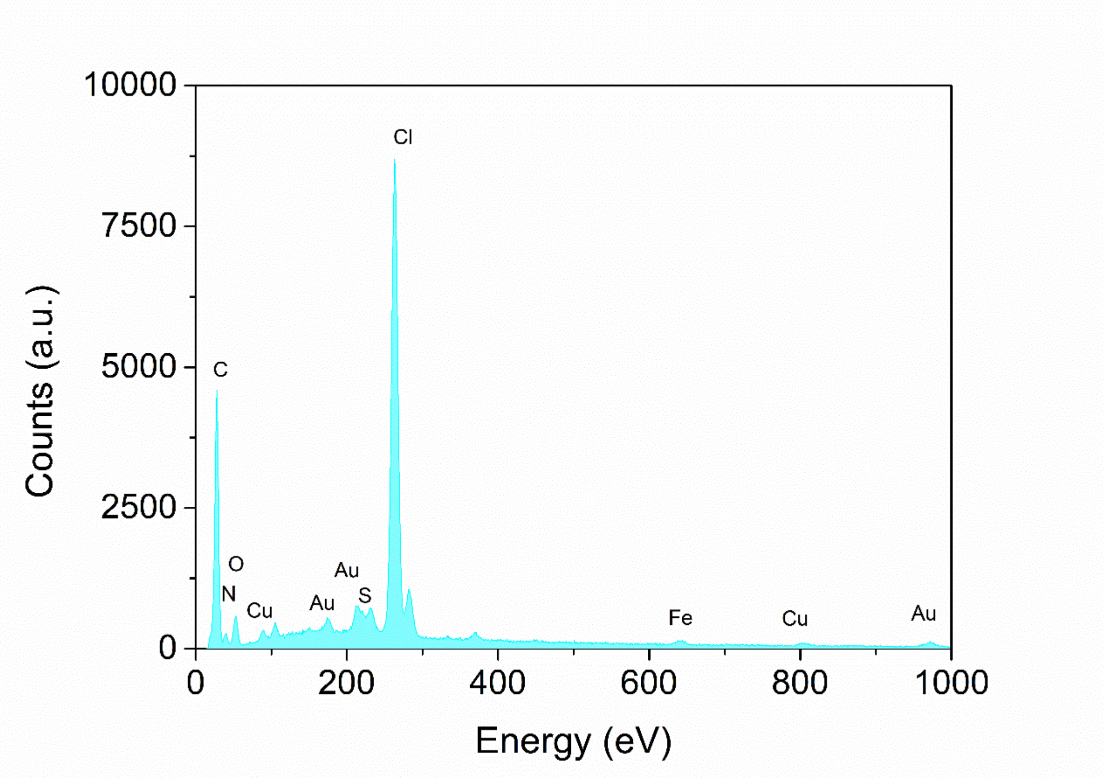

Figure S6. EDX spectra of the bCOF@$CuFeS_2$-SDS hybrid material.

Supporting Information

Table S1. EDX elemental analysis

Element	Wt %
**C K**	71.43
**N K**	2.18
**Au M**	2.19
**S K**	0.65
**Cl K**	12.70
**Fe K**	0.36
**Cu K**	0.48
**Total**	100.00

Supporting Information

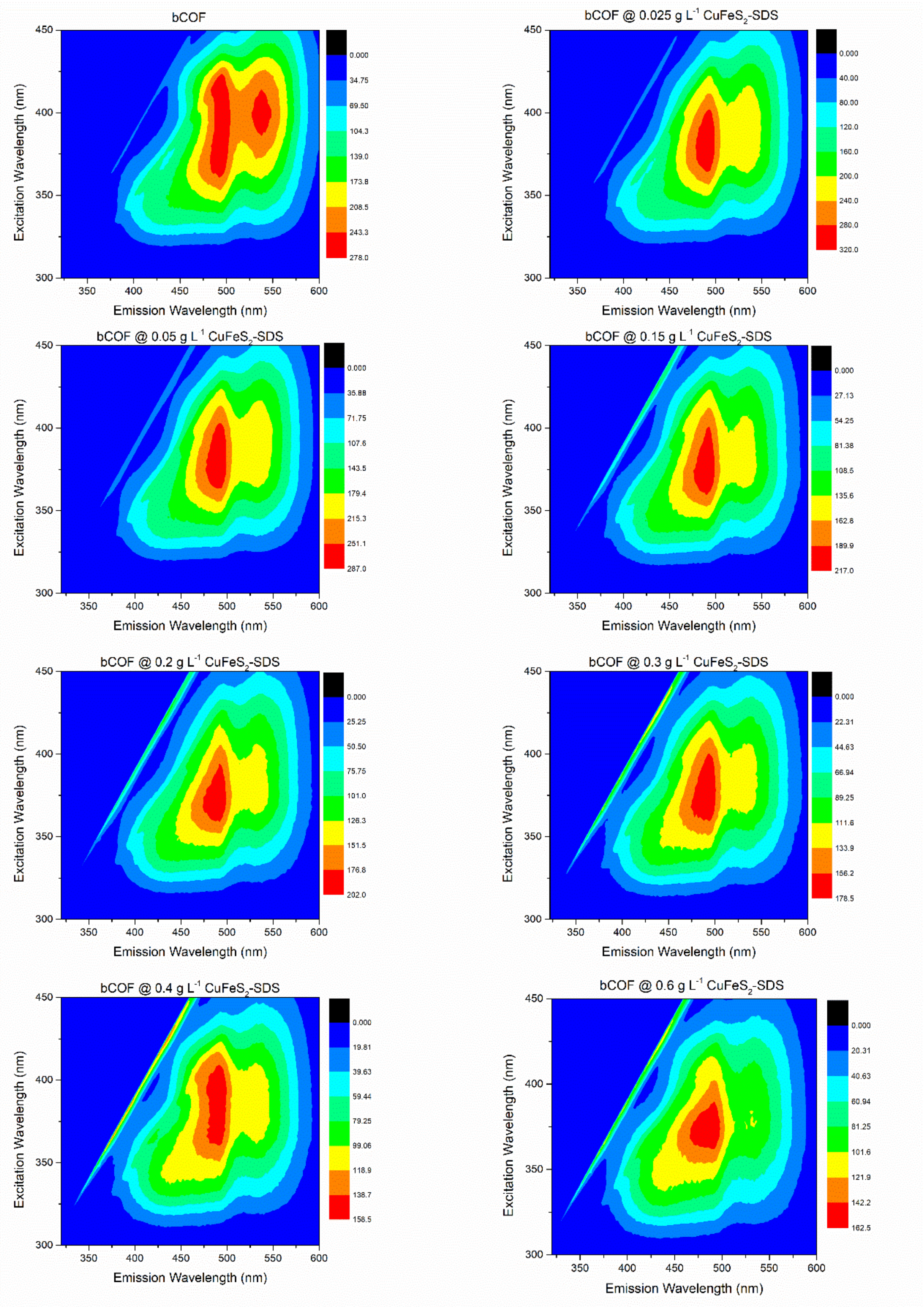

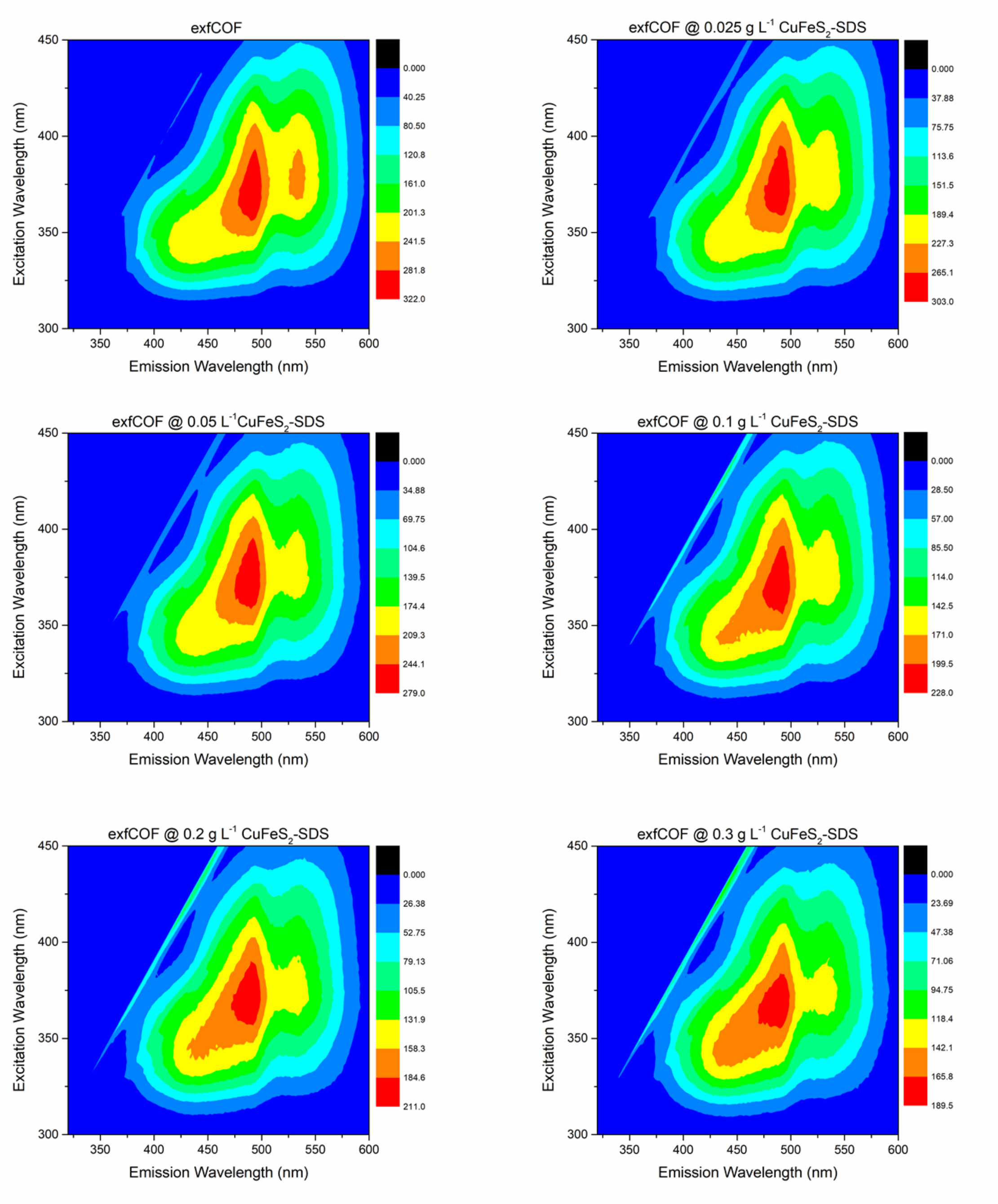

Figure S7. Excitation dependent photoluminescence mapping of the $CuFeS_2$-SDS titration in 0.2 g L^{-1} bCOF and exfCOF aqueous dispersions

a)

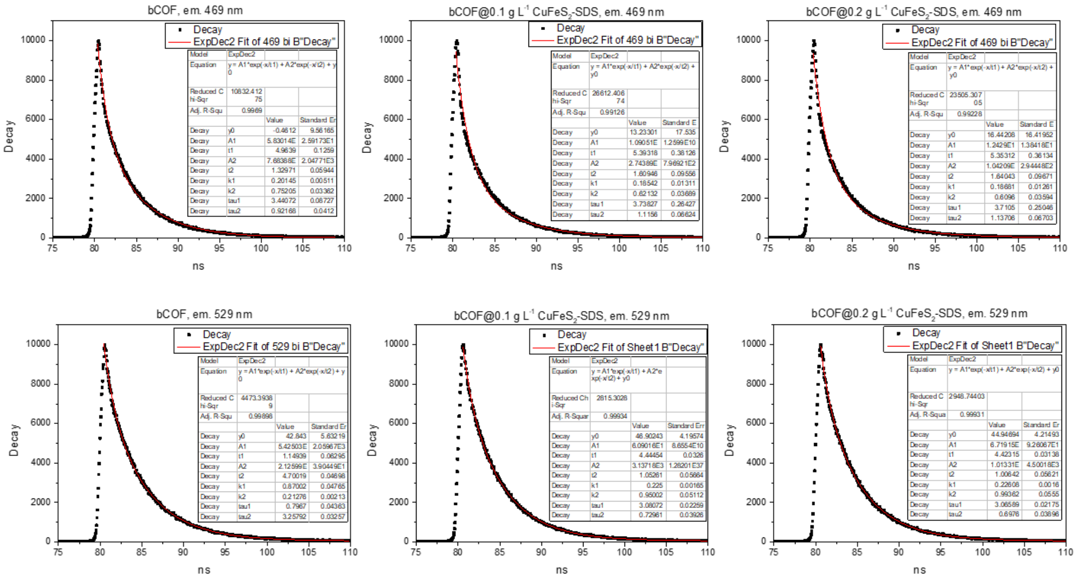

b)

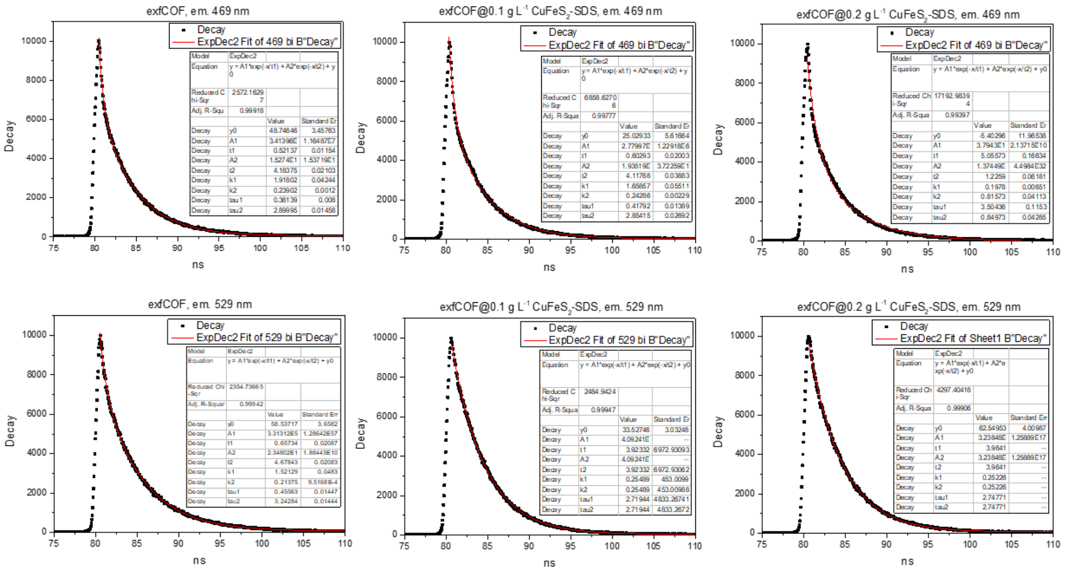

Figure S8. Time-resolved photoluminescence spectra and the bi-exponential fitting of a) bCOF and bCOF@$CuFeS_2$-SDS and b) exfCOF and exfCOF@$CuFeS_2$-SDS at the 469 nm and 529 nm emissions

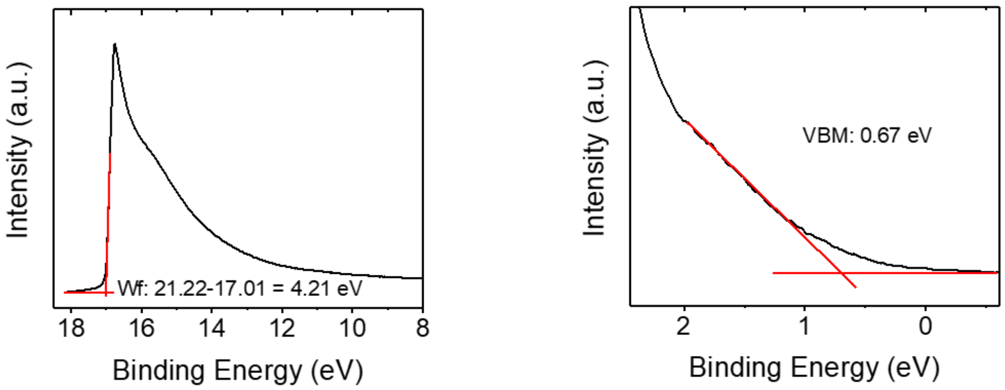

Figure S9. UPS measurements of $CuFeS_2$-OP nanoparticles on a FTO substrate.

bCOF@$CuFeS_2$-SDS

494 nm

Equation	y = a + b*x		
Weight	No Weighting		
Residual Sum of Squares	0.0414		
Pearson's r	0.97941		
Adj. R-Square	0.94906		
		Value	Standard Error
B	Intercept	1.02968	0.06282
	Slope	4996.32394	514.93408

536 nm

Equation	y = a + b*x		
Weight	No Weighting		
Residual Sum of Squares	0.03832		
Pearson's r	0.98669		
Adj. R-Square	0.96695		
		Value	Standard Error
C	Intercept	1.07285	0.06043
	Slope	6011.70449	495.37782

exfCOF@$CuFeS_2$-SDS

494 nm

Equation	y = a + b*x		
Weight	No Weighting		
Residual Sum of Squares	0.00198		
Pearson's r	0.9985		
Adj. R-Square	0.99626		
		Value	Standard Error
E	Intercept	0.98642	0.01375
	Slope	4116.48399	112.68093

536 nm

Equation	y = a + b*x		
Weight	No Weighting		
Residual Sum of Squares	0.01221		
Pearson's r	0.99375		
Adj. R-Square	0.98443		
		Value	Standard Error
F	Intercept	1.07341	0.03411
	Slope	4979.12525	279.61877

Figure S10. Parameters of the linear fitting at the F_0/F plots of bCOF@$CuFeS_2$-SDS and exfCOF@$CuFeS_2$-SDS (excitation:410 nm, emissions: 494 nm and 536 nm).

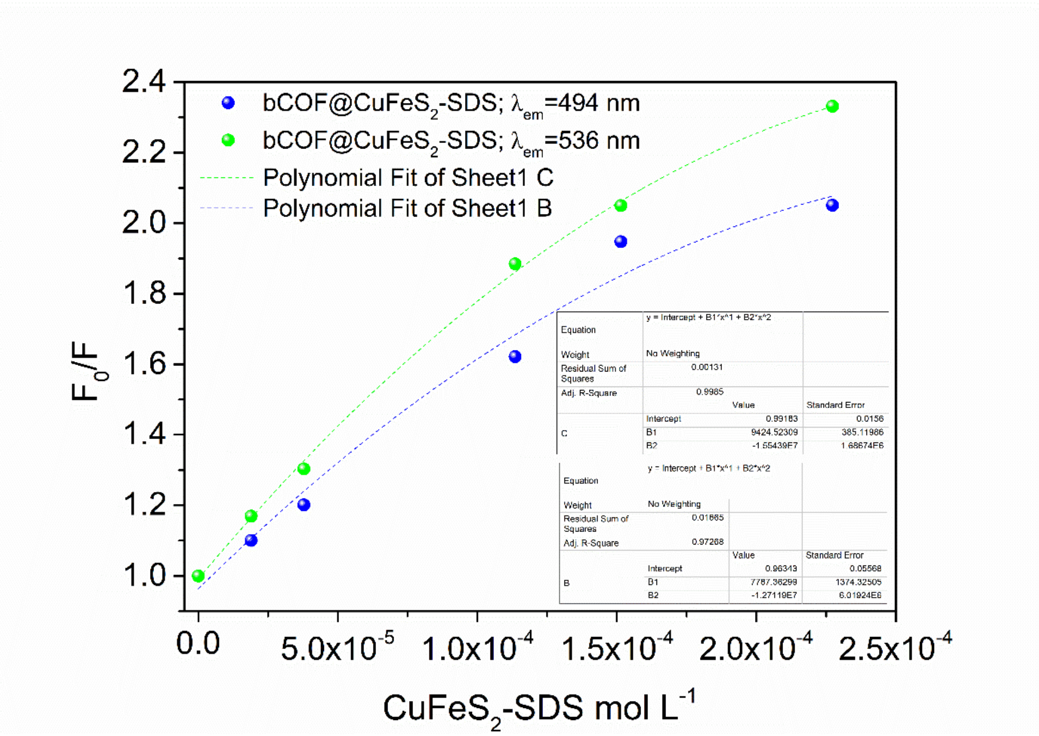

a)

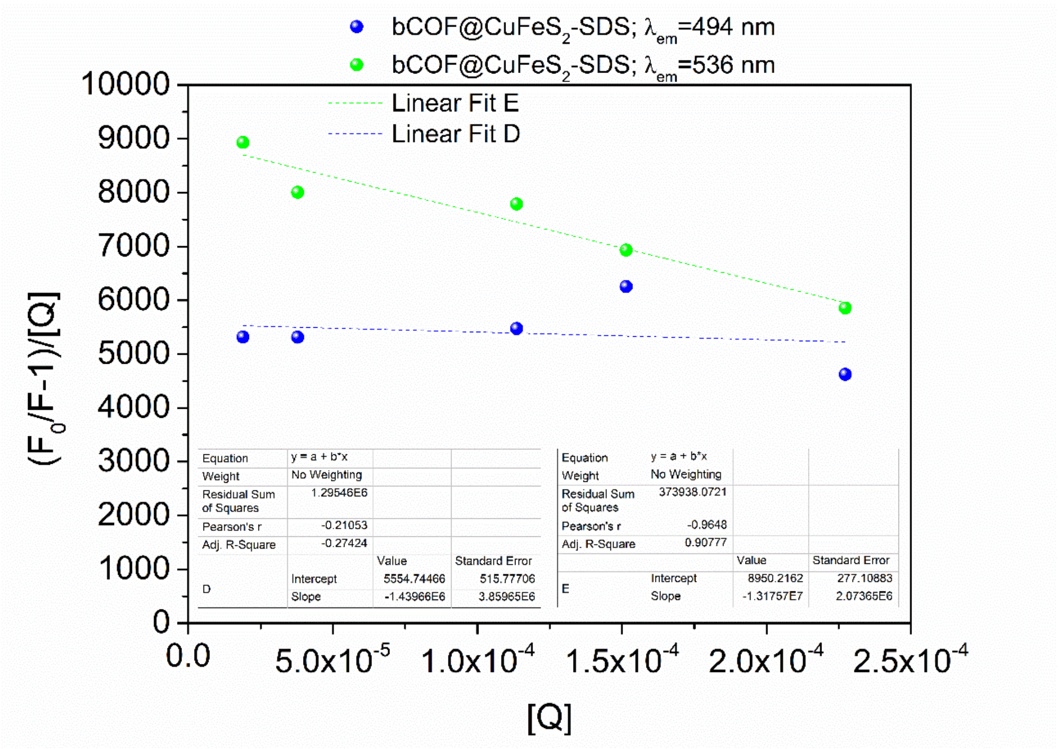

b)

Figure S11. a) The polynomial fitting and b) the parameters of the extended S-V, $(F_0/F\text{-}1)/[Q]$ with [Q] the concentration of the nanoparticles for bCOF@$CuFeS_2$-SDS.
